# Supplementary material for: Laser‐Induced Rashba Spin‐Orbit Torques in Multiferroic Semiconductor (Ge,Mn)Te
Source: Adv Sci (Weinh). 2026 Oct 28:e78355. Online ahead of print. doi: 10.1002/advs.78355 (PMC13717122; doi:10.1002/advs.78355)
Supplement: Supplementary file 1 [file ADVS-9999-e78355-s001.pdf]

Supplementary information

Laser-Induced Rashba Spin-Orbit Torques in Multiferroic Semiconductor (Ge,Mn)Te

*Zeynab Sadeghi, Tomáš Ostatnický, Eva Schmoranzerová, Jozef Kimák, Dominik Kriegner, Helena Reichlová, Lukáš Nádvorník, Gunther Springholz, J. Hugo Dil, Juraj Krempasky, and Petr Němec**

Contents

Supplementary Note 1. Magnetic and structural characterization of studied samples 2
Supplementary Note 2. Additional experimental data 4
2.1 Polarization dependence 4
2.2 Experiment below and above magnetic ordering temperature 5
2.3 Experiment in $Ge_{0.70}Mn_{0.30}Te$ reference sample 6
2.4 Time-resolved MO data within one hysteresis loop branch 6
2.5 Precession of magnetization 7
2.6 Angle-of-incidence dependence 7
2.7 Pump wavelength dependence 8
2.8 Magnetic origin of MO signals 9
2.9 Intensity dependence 11
Supplementary Note 3. Comparison of results measured in (Ge,Mn)Te with results reported for (Ga,Mn)As 13
Supplementary references 15

Supplementary Note 1. Magnetic and structural characterization of studied samples

Two 50-nm-thick (Ge,Mn)Te layers with different Mn concentrations, x_{Mn} = 15% and x_{Mn} = 30%, were grown by molecular beam epitaxy (MBE) on BaF_2 substrate at 280°C, using GeTe, Mn, and Te beam fluxes [S1]. A protective Al_2O_3 capping layer was deposited ex situ.

Structural characteristics

The samples were structurally characterized by X-ray diffraction angular scans (Fig. S1a). For both samples, a clear (Ge,Mn)Te peak corresponding to the rhombohedral structure is visible close to the BaF_2 (111) peak, confirming the high quality of the deposited layers. We further measured reciprocal space maps (RSMs) around the BaF2 (222) reflection at room temperature (Fig. S1b). For the sample with x_{Mn} = 15%, the (Ge,Mn)Te peak splits into at least two additional peaks due to the presence of ferroelectric domains oriented not only along the out-of-plane [111] direction, but also along the cubic body diagonals ⟨111⟩ [S1]. In contrast, no such splitting is observed for the sample with x_{Mn} = 30%, since this (Ge,Mn)Te layer is not ferroelectric at room temperature.

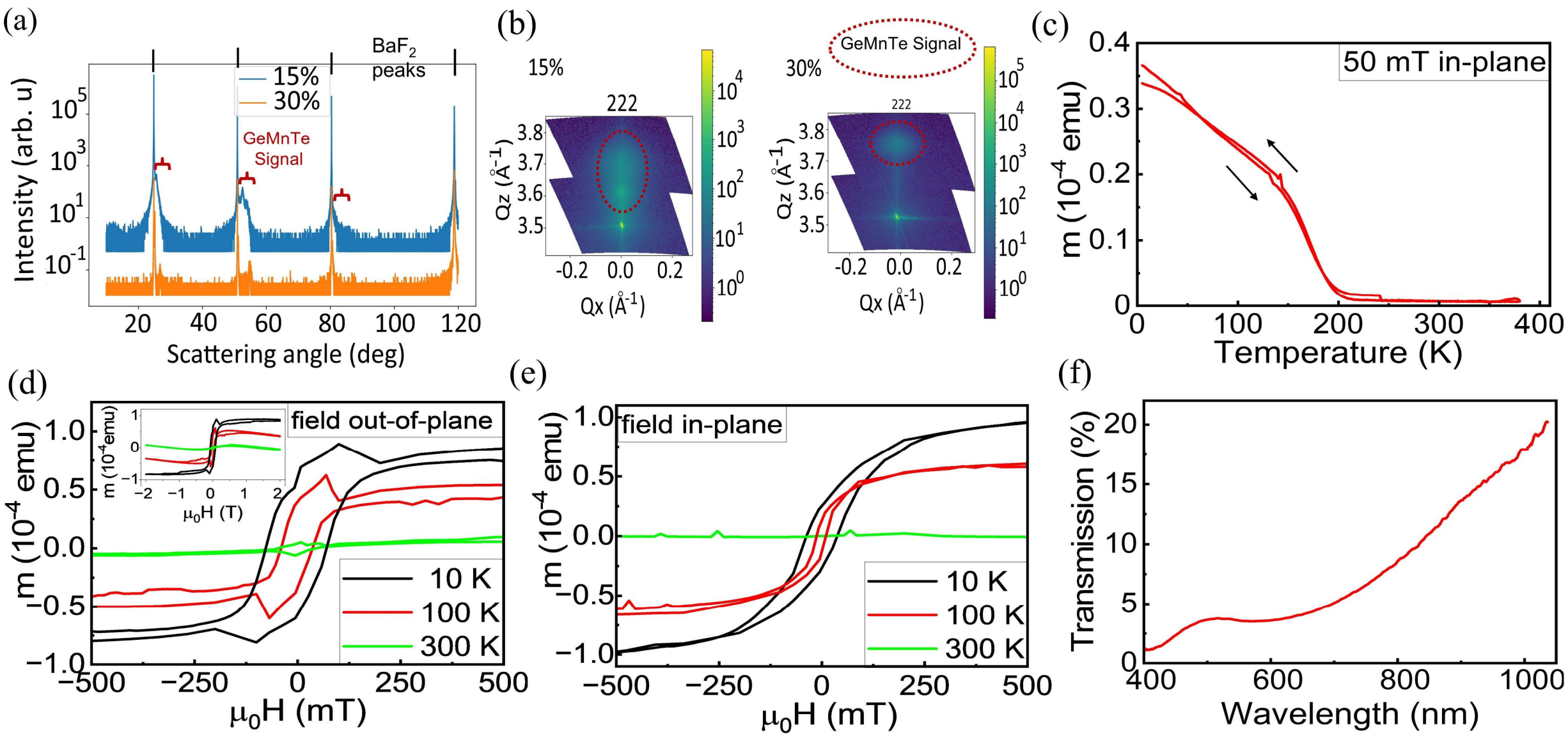

Fig. S1: Characterization of (Ge,Mn)Te samples. a, X-ray diffraction angular scans for samples with x_{Mn} = 15% (blue curve) and 30% (orange curve). Positions of the (Ge,Mn)Te peaks are indicated. **b**, Reciprocal space maps around the (222) BaF2 direction x_{Mn} = 15% (left panel) and 30% (right panel). Signal corresponding to (Ge,Mn)Te is indicated by red ellipse. **c**, Temperature dependence of magnetic moment *m*, measured with out-of-plane magnetic field of 50 mT, in the sample with 15 % of Mn. **d**, and **e**, Hysteresis loops in the out-of-plane (d) and in plane (e) directions. Inset in (d) shows the data in the full range of external magnetic fields. **f**, Transmission spectra of the samples with x_{Mn} = 15% at 15 K.

Magnetic characteristics

The magnetic properties of the 15% Mn sample were studied using a superconducting quantum interference device (SQUID) magnetometer. The temperature dependence of the magnetic moment, measured in an out-of-plane magnetic field of 50 mT, is shown in Fig. S1c.

The Curie temperature determined from this measurement is similar to the value obtained

from magneto-optical characterization (Figure 2e). From the hysteresis loops recorded with the magnetic field applied in the out-of-plane (OOP) (Fig. S1d) and in-plane (IP) (Fig. S1e) directions, we conclude that the sample exhibits dominant OOP magnetic anisotropy. The inset of Fig. S1c shows that the OOP magnetization saturates only at relatively large magnetic fields, around 1.5 T at 10 K.

Hole concentration

The hole concentration in our sample was estimated based on previous experiments reported in Ref. S1, Supplemental Fig. S3, where the hole concentration p is plotted as a function of the Mn concentration x_{Mn}. Those measurements were performed on nominally identical samples prepared in the same MBE chamber. We, therefore, estimate that for $x_{\mathrm{Mn}} = 15\%$, $p \approx 3 \times 10^{21}$ cm^{-3}.

Transmission spectra

Figure S1f shows the transmission spectrum of the sample with x_{Mn} = 15% at 15 K. This spectrum agrees with the spectral dependence expected for an indirect-bandgap semiconductor.

Supplementary Note 2. Additional time-resolved magneto-optical response

This supplementary note contains additional time-resolved magneto-optical results supporting the interpretation provided in the main paper.

2.1 Polarization dependence

Figure S2 compares the magnetic-field-dependent ellipticity signals measured for different polarizations of pump and probe pulses. The signal obtained for circularly polarized excitation closely follows the average response measured for the two orthogonal linear pump polarizations $\alpha = 45°$ and 135°, as shown in Fig. S2a. The difference between these two orthogonal linear pump polarizations is nearly field independent and very small compared with the main magneto-optical response, see Fig. S2b. This indicates that, within the sensitivity of this measurement, the field-dependent ellipticity signal is not strongly affected by the pump polarization.

A similar comparison was performed for the probe polarization dependence. The response measured for $\beta = 0°$ is almost identical to the average of the signals measured for $\beta = 45°$ and 135° (Fig. S2c), showing that the dominant field-dependent contribution is largely independent of the selected probe polarization. The existing difference between the data measured for $\beta = 45°$ and 135° (Fig. S2d) can be attributed to Voigt magneto-optical effect, which is quadratic-in-magnetization.

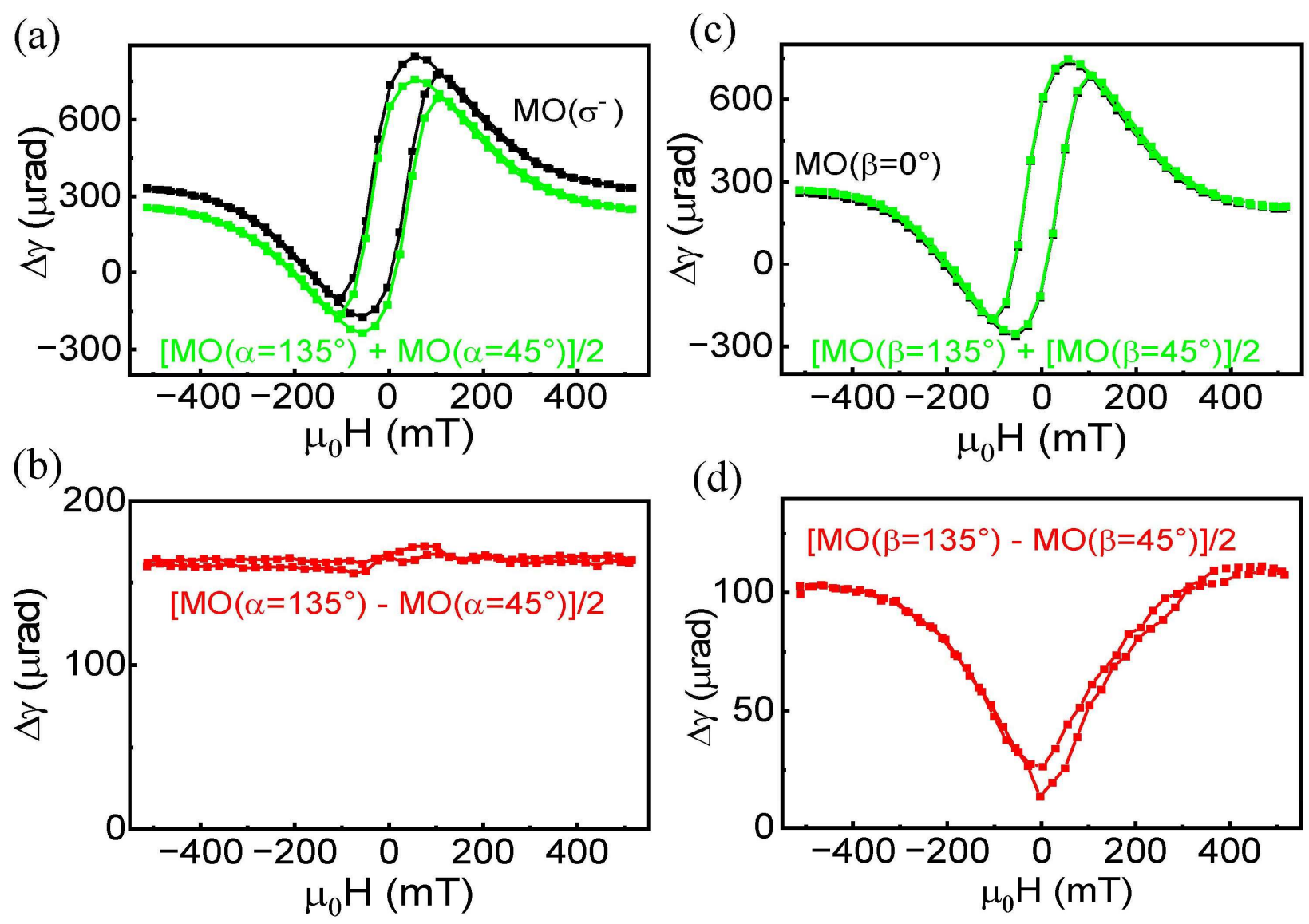

Fig. S2: Pump- and probe-polarization dependences. **a**, Field-dependent ellipticity $\Delta\gamma$ measured for $\Delta t = 20$ ps for circular pump polarization $\boldsymbol{\sigma}^{-}$ is compared with the average of signals measured for linear pump polarizations $\alpha = 45°$ and 135°. **b**, Difference between the signals measured for pump polarizations $\alpha = 45°$ and 135°. **c**, Signal measured for probe polarization $\beta = 0°$ is compared with the average of the signals measured for probe polarizations $\beta = 45°$ and 135°. **d**, Difference between the signals measured for probe polarizations $\beta = 45°$ and 135°. Measurements were performed in the standard geometry for pump fluence 1.3 I_{th}, wavelength 820 nm, probe wavelength 633 nm, AOI = -5°, $\Theta_H = -15°$.

2.2 Experiment below and above magnetic ordering temperature

At 15 K, the magnetic-field-dependent ellipticity loops show a clear hysteretic response for all pump polarizations, see Fig. S3a. The overall signal contains a polarization-dependent field-independent offset: the largest positive background is observed for α = 135°, while the largest negative background is observed for α = 45°. As discussed in the Methods section of the main text, we separate the dynamic into two components, $\Delta\gamma^{\mathrm{dif}}$ and $\Delta\gamma^{\mathrm{aver}}$. The differential part $\Delta\gamma^{\mathrm{dif}}$ contains the part of the signal that changes sign when the magnetic field is reversed. In contrast, the $\Delta\gamma^{\mathrm{aver}}$ component contains the part of the signal that does not change the sign under the field reversal.

Using this separation procedure, the $\Delta\gamma^{\mathrm{dif}}$ ellipticity dynamics measured at 500 mT are nearly identical for all pump polarizations, indicating that the magnetic part of the transient MO response does not depend strongly on the pump polarization (Fig. S3b). In contrast, the $\Delta\gamma^{\mathrm{aver}}$ component shows a pronounced pump-polarization dependence, following the same trend as the offset observed in the hysteresis loops (Fig. S3c).

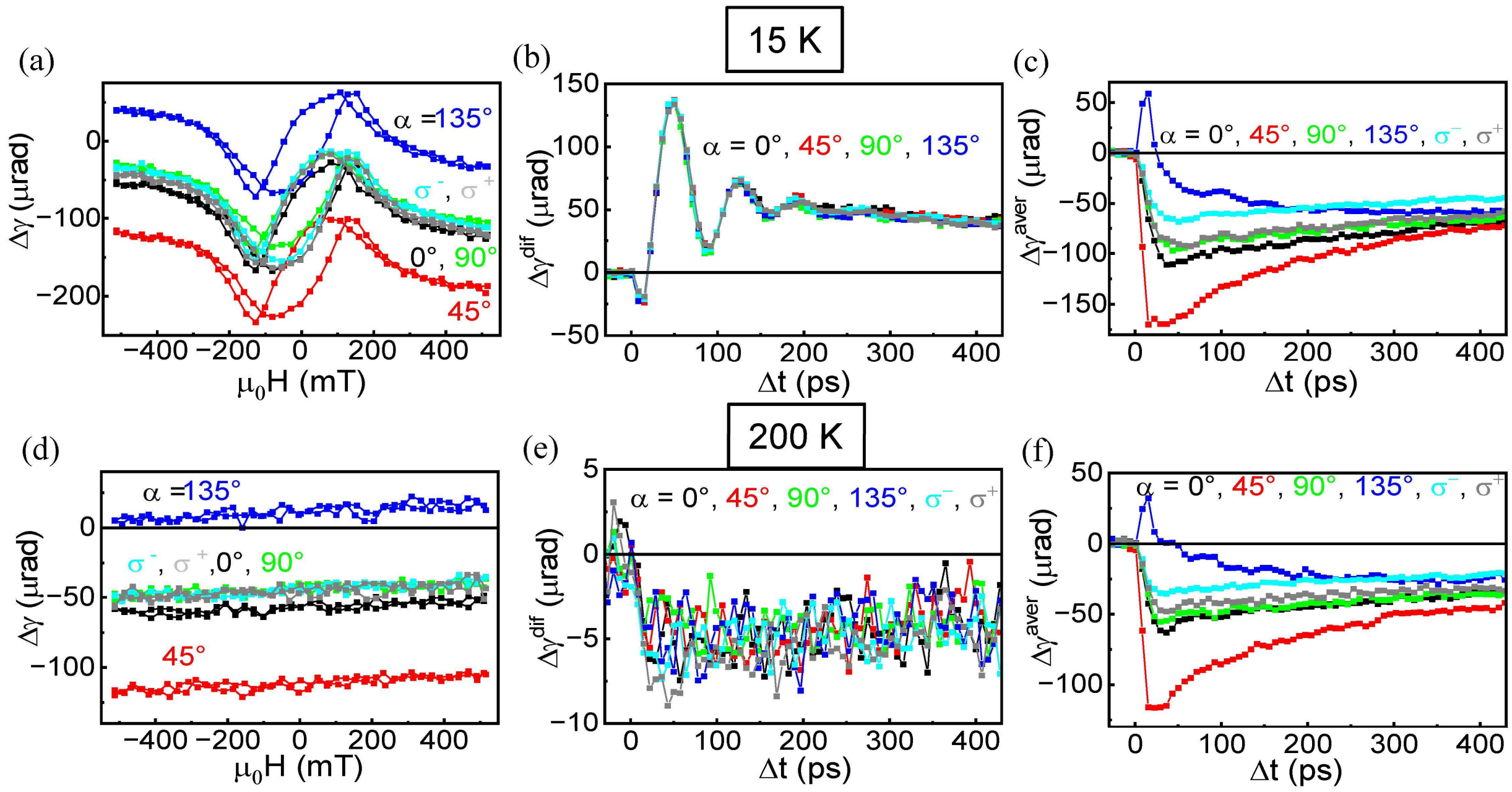

Fig. S3: Pump-polarization dependence of MO signals at 15 K and 200 K. a, Magnetic-field-dependent ellipticity hysteresis loops measured at 15 K for Δt = 20 ps for different linear pump polarizations α = 0°, 45°, 90°, and 135°, and for circularly polarized excitations σ− and σ+.
b, and **c**, Dynamics of signals $\Delta\gamma^{dif}$ (b) and $\Delta\gamma^{aver}$ (c), see Methods for their definitions, measured at 15 K for 500 mT as a function of time delay for different pump polarizations. **d**, **e**, and **f**, Same as a, b, and c, respectively, measured at 200 K. All measurements were performed in the standard geometry for pump wavelength 820 nm, probe wavelength 633 nm, AOI = 0°, Θ_H = -10°.

The measurements at 200 K, which is above the magnetic transition temperature in the studied sample, provide a direct evidence how the measured signals change due to the quenched magnetic ordering in the sample. At this temperature, the hysteretic field-dependent signal is completely absent (Fig. S3d), and the component $\Delta\gamma^{dif}$ is reduced to a very weak signal (Fig. S3e). This confirms that the $\Delta\gamma^{dif}$ signal is connected with the magnetic order. On the other hand, the component $\Delta\gamma^{aver}$ is not affected significantly by the temperature increase and still shows pump-polarization-dependent signals (Fig. S3f). This suggests that the $\Delta\gamma^{aver}$ signal is mainly related to a pump-induced non-magnetic optical background rather than to the magnetic signal detected through the field-even MO Voigt effect.

2.3 Experiment in $Ge_{0.70}Mn_{0.30}Te$ reference sample

Reference time-resolved pump–probe experiments were performed in the $Ge_{0.70}Mn_{0.30}Te$ film. As shown in Figure S4a, the magnetic-field-dependent ellipticity measured for different pump polarizations show strong suppression of the hysteretic response. Similarly, the pump-polarization-independent $\Delta\gamma^{dif}$ signal (Fig. S4b) is rather small in this sample. On the other hand, there still a rather pronounced pump-polarization-dependent offset in the hysteresis loops (Fig. S4b) together with a rather large pump-polarization-dependent $\Delta\gamma^{aver}$ signal (Fig. S4c).

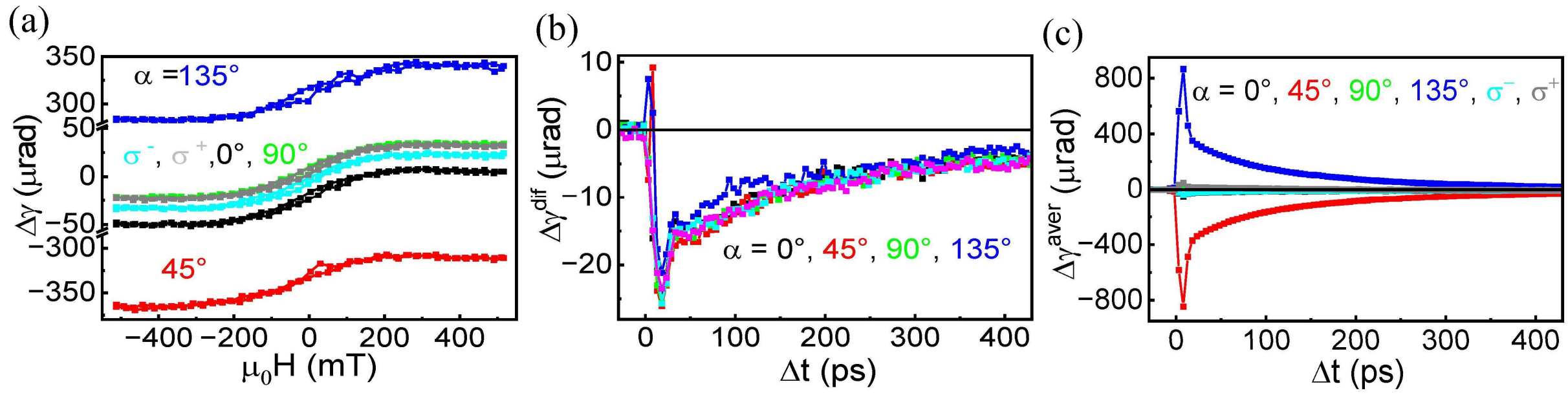

Fig. S4: Pump-polarization dependence of MO signals measured at 15 K in $Ge_{0.70}Mn_{0.30}Te$. **a**, Magnetic-field-dependent ellipticity hysteresis loops measured for $\Delta t = 20$ ps for different linear pump polarizations $\alpha = 0°, 45°, 90°$, and 135°, and for circularly polarized excitations σ− and σ+. **b**, and **c**, Dynamics of signals $\Delta\gamma^{dif}$ (b) and $\Delta\gamma^{aver}$ (c) measured for 500 mT as a function of time delay for different pump polarizations. All measurements were performed in the standard geometry for pump wavelength 820 nm, probe wavelength 633 nm, AOI = 0°, $\Theta_H = -10°$.

2.4 Time-resolved MO data within one hysteresis loop branch

Figures S5b and S3c show evolution of time-resolved signals when the magnetic field is swept from positive to negative fields within one hysteresis loop branch (see Fig. S5a).

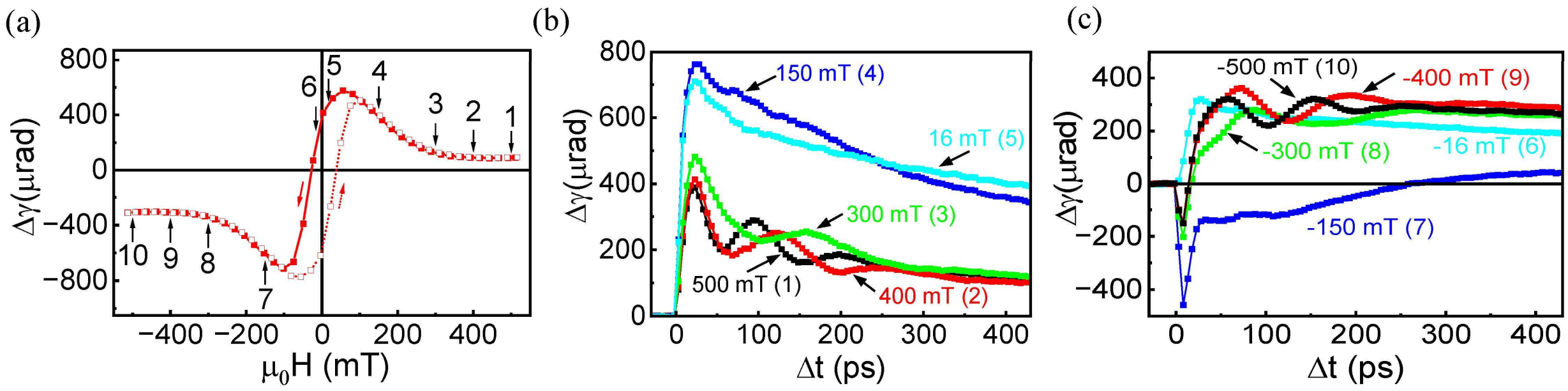

Fig. S5: Magnetic-field dependence of the time-resolved MO signal. **a**, Magnetic-field-dependent ellipticity hysteresis loops measured for $\Delta t = 2$ ps; the numbers by the vertical arrows indicate the measurements sequence, starting from +500 mT (1) and ending at −500 mT (10).
b, and **c**, Dynamics of $\Delta\gamma$ measured at fields depicted in a. All measurements were performed in the standard geometry for pump fluence 1.3 I_{th}, wavelength 820 nm, probe wavelength 633 nm, AOI = -5°, $\Theta_H = -15°$.

2.5 Precession of magnetization

Figure S6 shows the magnetic-field dependence of $\Delta\gamma^{dif*}$ signal, corresponding to a difference of MO signals measured at depicted fields within one branch of the hysteresis loop (see Figure S5a and Methods). The squares are the experimental data and the red solid lines are fits. The dynamics were fitted using equation 3 described in the Methods section where the first term describes the damped oscillatory response and the second term accounts for the non-oscillatory background. The fits reproduce the experimental data well for all three magnetic fields. The oscillation period becomes longer as the magnetic field decreases, showing that the precession frequency is reduced at lower fields (Fig. S6d). The values of other fitting parameters are shown in Figs. S6e and S6f. The oscillation amplitude A is nearly field-independent while the background amplitude B decreases for higher fields. The precession phase δ changes only weakly with magnetic field, and the decay time $\boldsymbol{\tau_1}$ shows a moderate decrease at higher fields.

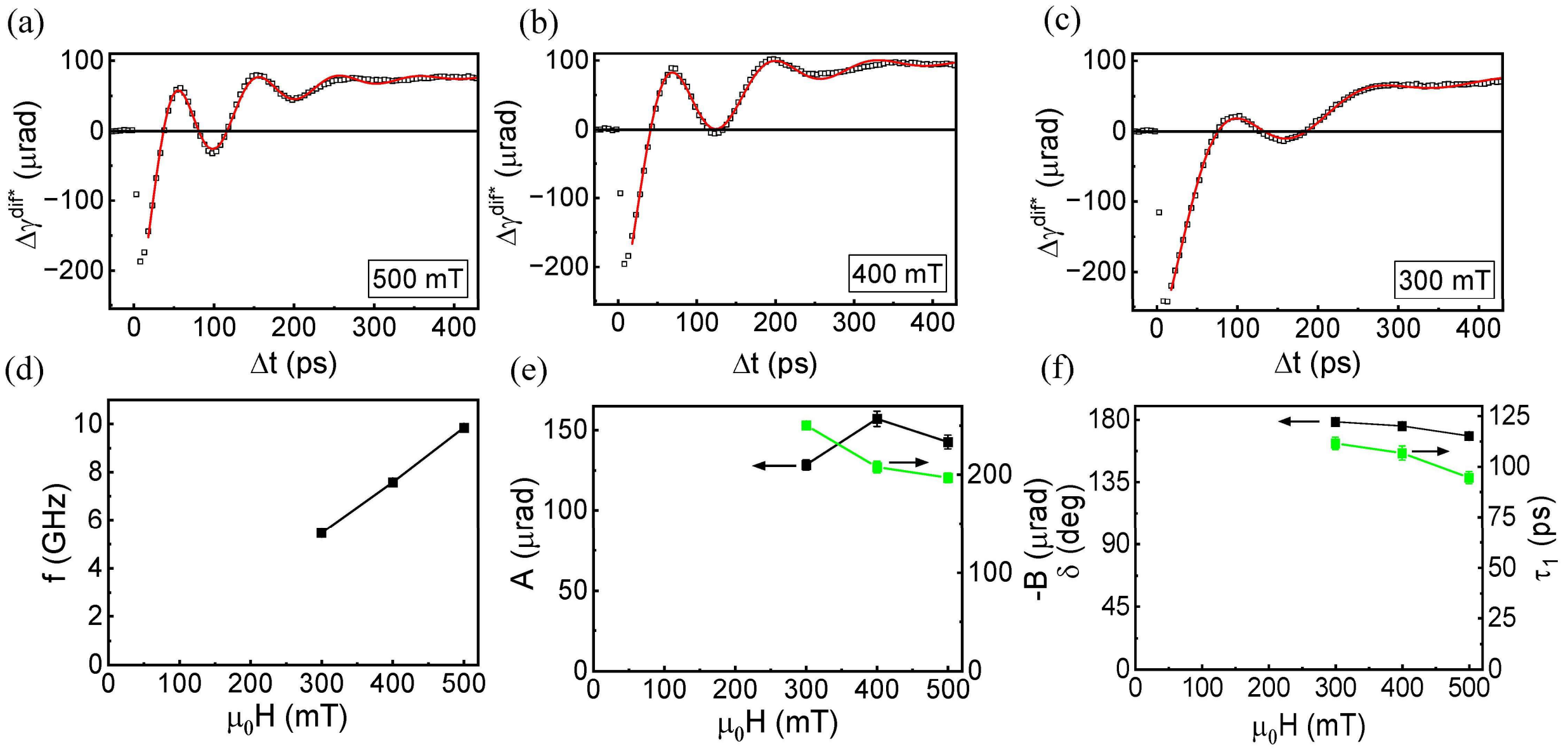

Fig. S6: Time-resolved MO signals for selected magnetic fields. a, **b**, and **c**, Dynamics of $\Delta\gamma^{dif*}$ measured for 500 mT, 400 mT, and 300 mT, respectively (points). Red solid lines show the fits by Equation 3 (see Methods). **d**, **e**, and **f**, Magnetic-field dependence of the fitted parameters describing the measured signals: frequency f (d), oscillation amplitude A together with the background amplitude B (e), and phase δ together with the decay time τ_1 (f). All measurements were performed in the standard geometry for pump fluence 1.3 I_{th}, wavelength 820 nm, probe wavelength 633 nm, AOI = -5°, Θ_H = -15°.

2.6 Angle-of-incidence dependence

Figure S7 shows how the obtained signals depend on the probe angle of incidence (AOI). In the experiment, the angle of incidence was varied by rotating the sample, which also changed the angle between the applied magnetic field and the sample plane (see inset in Fig. 1a). The three representative geometries are shown in Figs. S7a, S7b and S7c for AOI = −5°, 0° and +5°, respectively. The corresponding magnetic-field-dependent ellipticity loops are shown in Fig. S7d for several several values of AOI. Clearly, the shape and amplitude of the hysteresis loops change with AOI, indicating that the measured magneto-optical response is sensitive to the experimental geometry. The dynamics of $\Delta\gamma^{dif}$ and $\Delta\gamma^{aver}$ are shown in Fig. S7e and Fig. S7f, respectively. For all measured angles, the $\Delta\gamma^{dif}$ signal contains a damped oscillatory contribution, although its amplitude and frequency vary with AOI, see Figs. S7g, S7h and S7i. On the other hand, the $\Delta\gamma^{dif}$ signal does not depend on AOI significantly.

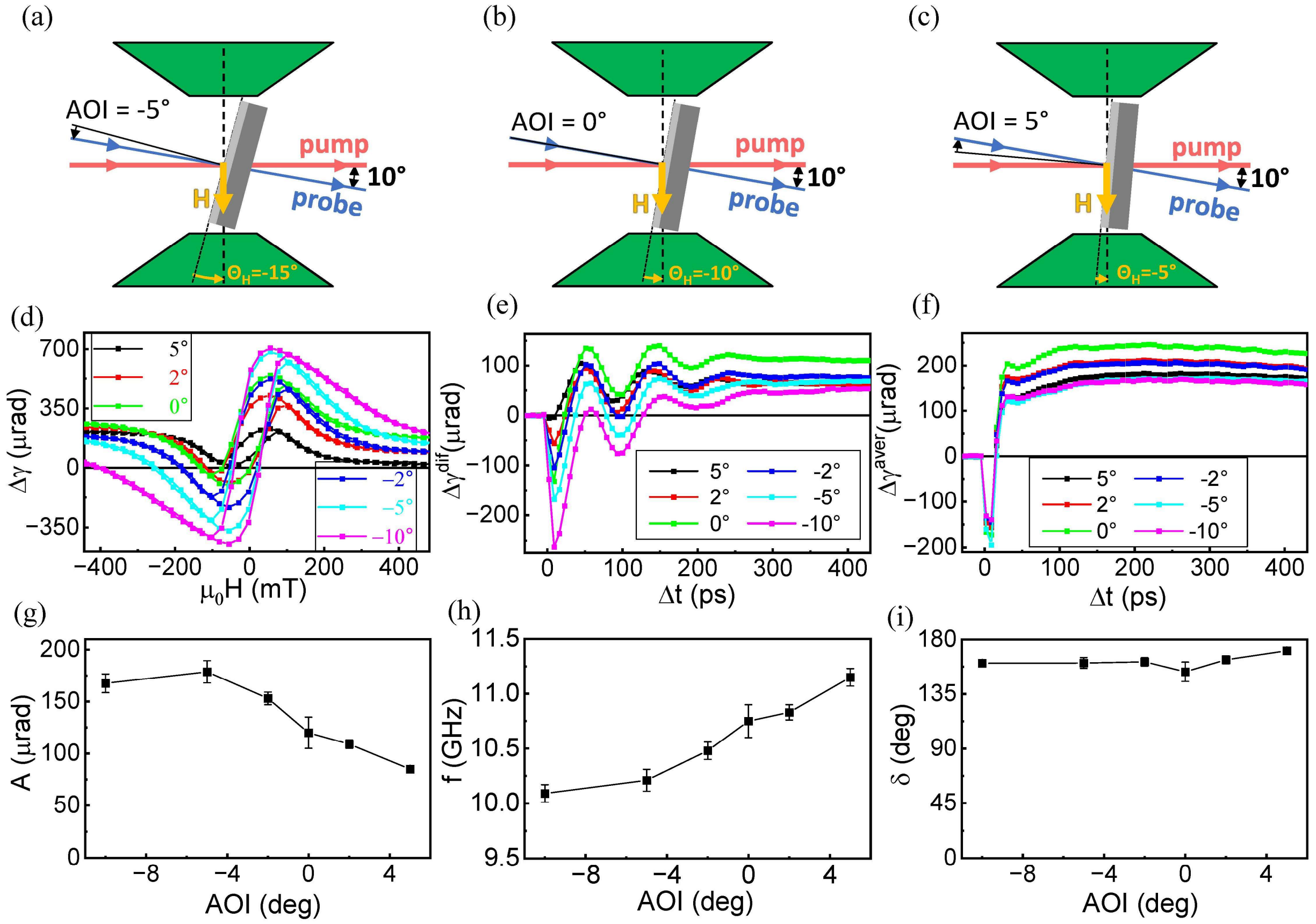

Fig. S7: Influence of the angle of incidence (AOI) on the measured signals.
a, **b**, and **c**, Schematic depiction of experimental geometries for different values of AOI and the resulting magnetic-field tilt angles θ_H. a: AOI = −5°, θ_H = −15°, b: AOI = 0°, θ_H= −10°, c: AOI = +5°, θ_H = −5°. **d**, Magnetic-field-dependent ellipticity hysteresis loops measured for Δt = 20 ps at depicted values of AOI. **e**, and **f**, Dynamics of signals $\Delta\gamma^{dif}$ (e) and $\Delta\gamma^{aver}$ (f) obtained at depicted values of AOI. **g**, **h**, and **i**, Dependence of the fitting parameters, used to describe the magnetization precession in e, by Equation 3, on AOI: precession amplitude A (g), frequency f (h), and phase δ (i). All measurements were performed in the standard geometry for pump fluence 1.3 I_{th}, wavelength 820 nm, probe wavelength 633 nm.

2.7 Pump-wavelength dependence

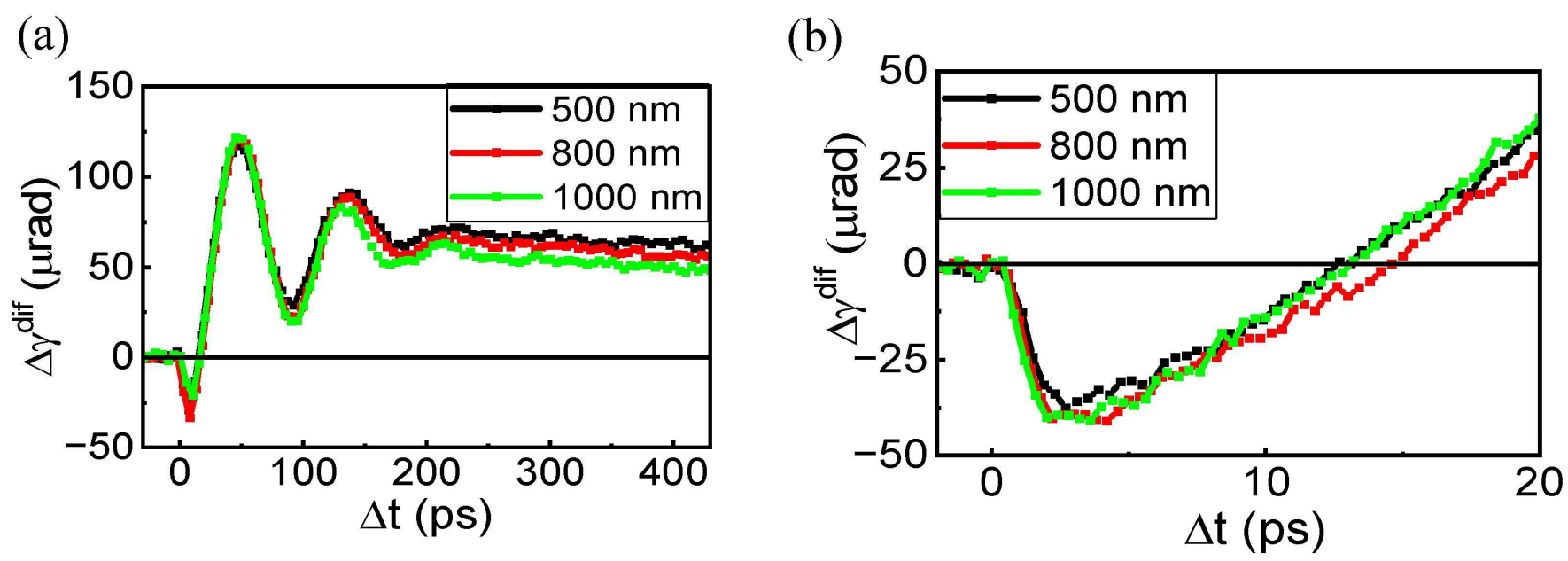

Fig. S8: Pump-wavelength dependence of the $\Delta\gamma^{dif}$ signal. **a**, and **b**, Dynamics of $\Delta\gamma^{dif}$ signal measured for pump wavelengths of 500, 800, and 1000 nm over the intermediate (a) and short (b) time ranges, respectively. All measurements were performed in the standard geometry probe wavelength 633 nm, AOI = 0°, applied magnetic field 500 mT. At each wavelength, the pump fluence was adjusted to obtain a fixed value of the transient transmission, which eliminates the role of spectrally dependent absorption coefficient in the sample.

Figure S8 shows the dependence of the $\Delta\gamma^{dif}$ signal dynamics on the pump wavelength. For all pump wavelengths, the dynamics show very similar behavior, including the initial rise, the damped oscillatory response, and the subsequent slow background evolution. This indicates that the magnetic dynamics are not strongly affected by changing the pump wavelength within the measured range.

2.8 Magnetic origin of MO signals

Magneto-optical (MO) investigation of the magnetization state and its dynamics relies on measuring changes in the polarization state of probe light – specifically, its rotation and change in ellipticity – induced by interaction with a sample. In principle, there are two contributions to the measured signals: one arises from symmetry breaking due to magnetization, and the other from non-magnetic optical anisotropy of the sample. In this article, we focus on the $\Delta\gamma^{dif}$ and $\Delta\beta^{dif}$ signals, which correspond to the signal components that change sign when the direction of the external magnetic field is reversed (see Eq. (1) in Methods). Therefore, this data processing completely removes the non-magnetic component from the raw data, leaving only the MO response of the sample.

Nevertheless, pump-induced MO signals are sensitive not only to the magnetic dynamics of the system under study but also to laser-induced changes in the complex refractive index. This contribution is often referred to as the optical part of the MO signal. To determine whether the magnetic or optical part dominates in the measured dynamical data, both the pump-induced changes in rotation $\Delta\beta$ and ellipticity $\Delta\gamma$ should be measured, and their dynamics compared [S2].

In the static case, when only the Faraday effect is present, rotation β and ellipticity γ could be described as

$$\beta = f_\beta M, \tag{S1}$$

$$\gamma = f_\gamma M, \tag{S2}$$

where f_β and f_γ are optical ”constants”, which dependent on material electronic properties and light wavelength, and M is magnetization. If a pump pulse induces a material change in the studied sample, it leads to a time-dependent change of polarization rotation $\Delta\beta$(t) and ellipticity $\Delta\gamma$(t). Since both optical and magnetic parts could be modified by the pump pulse, we have

$$\Delta\beta(t) \approx \Delta f_\beta(t) M + f_\beta \Delta M(t)\ , \tag{S3}$$

$$\Delta\gamma(t) \approx \Delta f_\gamma(t) M + f_\gamma \Delta M(t)\ . \tag{S4}$$

The first terms on the right-hand sides are the optical parts, the second terms are the magnetic parts. One can see that in general case the dynamics of rotation and ellipticity does not need to be equal. However, if the magnetic parts dominate, i.e., if $\Delta f_\beta(t) M \ll f_\beta \Delta M(t)$ and $\Delta f_\gamma(t) M \ll f_\gamma \Delta M(t)$, then

$$\Delta\gamma(t) \approx \frac{f_\gamma}{f_\beta} \Delta\beta(t) \approx f_\gamma \Delta M(t)\ . \tag{S5}$$

This means that for $\Delta\beta(t)$ and $\Delta\gamma(t)$ to represent magnetic dynamics, the dynamics of rotation and ellipticity must be equal, up to a multiplicative constant. Similar analysis shows that measurements of ellipticity (or rotation) dynamics for various probe wavelengths must lead to the same dynamics, up to a multiplicative constant.

In Figure S9 the ellipticity $\Delta\gamma^{dif}$ and rotation $\Delta\beta^{dif}$ signals in the standard measurement geometry are compared. In Figure S9a and S9b the dynamics measured at probe wavelengths of 450 nm and 550 nm are shown, respectively. To enable a direct comparison of these dynamics, the rotation dynamics were multiplied by the depicted scaling factors. At both wavelengths, the rotation and ellipticity signals follow very similar time dependences, including the initial rise and the following relaxation. This shows that both these magneto-optical detection channels are sensitive to the same pump-induced change of magnetic properties in GeMnTe sample for time delays ≥ 1 ps, where the signals start to decay. This also reveals that the pump-induced change of magnetic properties is fully completed in less than 1 ps.

For comparison, the transient transmission $\Delta T/T$ measured at several probe wavelengths is shown in Fig. S9c. On time scales up to ≈ 10 ps, the signal strongly depends on the probe wavelength, in both sign and amplitude, reflecting the relaxation of photoexcited carriers within the band structure and their trapping at defect states. This leads to a time- and wavelength-dependent evolution of the complex refractive index of the excited film. This effect is most pronounced for time delays up to ≈ 1 ps, when the carriers lose their excess energy, which is why optical effects dominate in Equations (S3) and (S4) in this time range.

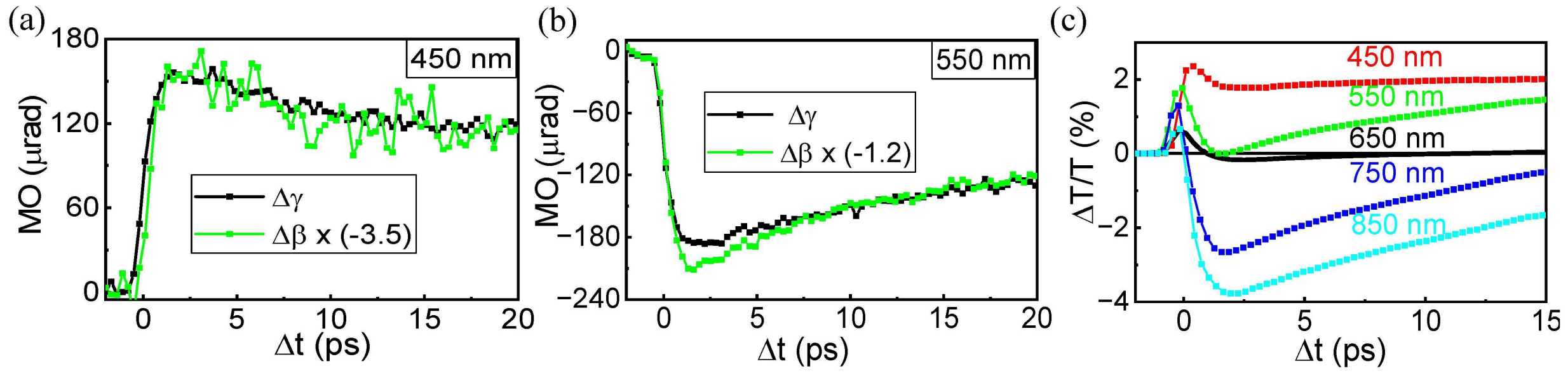

Fig. S9: Comparison of rotation and ellipticity dynamics in the standard geometry.
a, Comparison of the ellipticity $\Delta\gamma^{dif}$ and rotation $\Delta\beta^{dif}$ signals measured at a probe wavelength of 450 nm. The rotation signal is multiplied by a factor −3.5 to enable a direct comparison with the ellipticity data. **b**, Same as in a, for a probe wavelength of 550 nm; the rotation signal is multiplied by a factor −1.2. **c**, Transient transmission $\Delta T/T$ measured for different probe wavelength. Pump wavelength 1000 nm, AOI = 0°, applied magnetic field 16 mT.

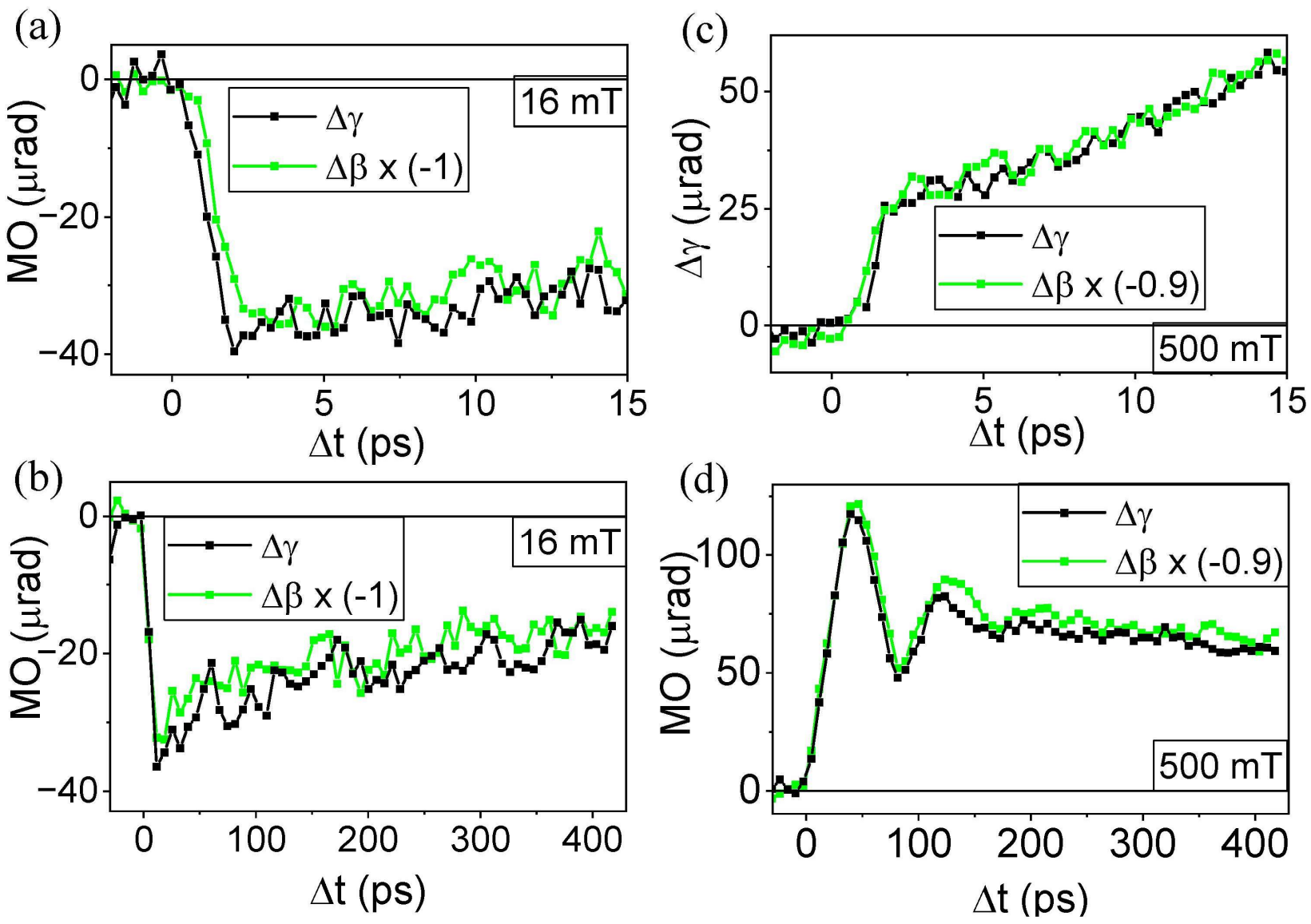

Fig. S10: Comparison of rotation and ellipticity dynamics in the concentric geometry.
a, and **b**, Comparison of the short (a) and intermediate (b) dynamics of ellipticity $\Delta\gamma^{dif}$ and rotation $\Delta\beta^{dif}$ signals measured at 16 mT. **c**, and **d**, Same as a, and b, for 500 mT. Pump wavelength 800 nm, probe wavelength 532 nm.

Figure S10 compares the ellipticity $\Delta\gamma^{dif}$ and rotation $\Delta\beta^{dif}$ signals in the concentric geometry at low and high magnetic fields. As for the standard geometry, this indicates that both magneto-optical detection channels are sensitive to the same pump-induced change of magnetic properties in GeMnTe sample for time delays ≥ 1 ps. Moreover, these results also verify that the pump-induced change of magnetic properties is fully completed in ≤ 1 ps.

2.9 Intensity dependence

Figure S11 shows how the magnetic-field-dependent ellipticity loops evolve with the pump intensity in the standard geometry. When the pump intensity is increased above the threshold fluence I_{th}, the loop amplitude grows rapidly, indicating a pronounced enhancement of the transient magneto-optical response. However, the measured signal approaches saturation at higher pump intensities.

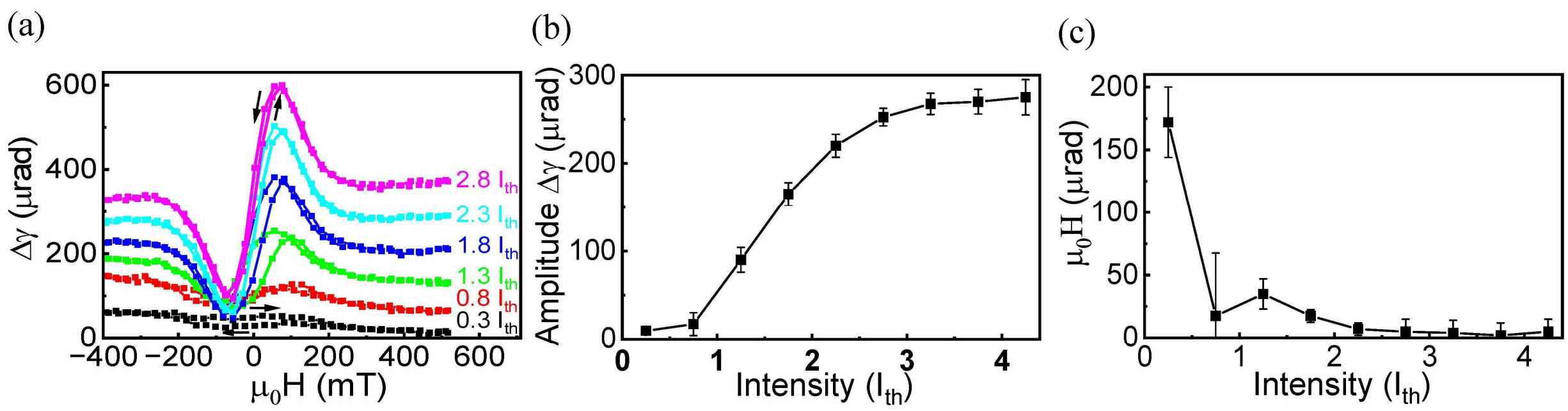

Fig. S11: Pump-intensity dependence in the standard geometry. a, Magnetic-field-dependent ellipticity hysteresis loops measured for Δt = 20 ps at different pump intensities. **b**, and **c**, Pump-intensity dependence of the hysteresis-loop amplitude (b) and the coercive field (c) extracted from data shown in panel a. , Pump wavelength 1 000 nm, probe wavelength 670 nm, AOI = 0°.

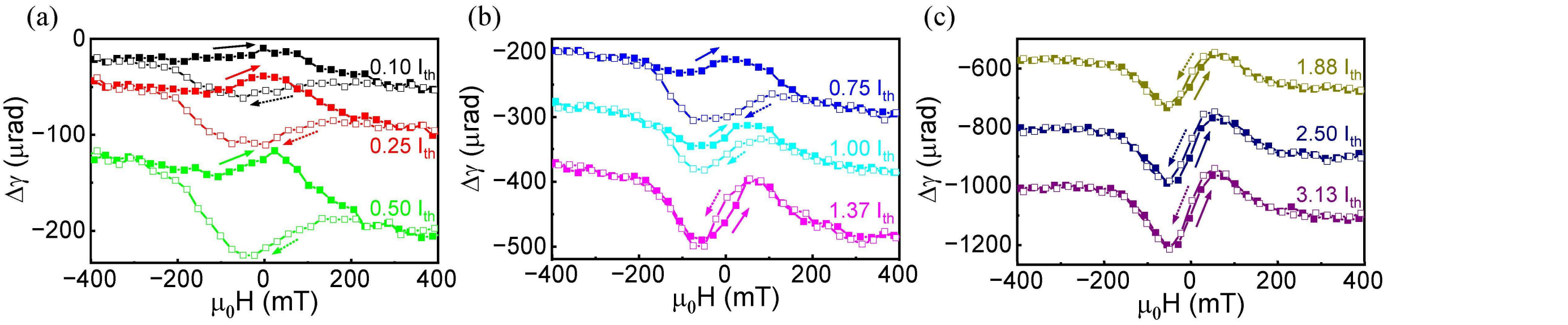

Fig. S12: Pump-intensity dependence in the concentric geometry. a, **b**, and **c**, Magnetic-field-dependent ellipticity hysteresis loops measured for $\Delta t = 20$ ps at different pump intensities. Pump wavelength 800 nm, probe wavelength 532 nm.

Figure S12 shows how the magnetic-field-dependent ellipticity loops evolve with pump intensity in the concentric geometry. At the lowest intensity of 0.1 I_{th} , the hysteresis loop closely resembles the static loops, which are a monotonic function of the external field at all temperatures (see Figure 2d). However, as the intensity increases, the character of the loops changes considerably. In particular, as the laser fluence approaches the threshold fluence I_{th}, the MO signals become a strongly nonmonotonic function of the applied magnetic field. Moreover, the coercive fields are strongly reduced above I_{th} (Figure 6f). To express the change in loop character, Figure 6d shows the amplitude of the hysteresis loops, with the sign defined as follows when changing the field from its negative extreme. For monotonic functions of the external field, resembling the static case (Figure 2d), the sign of the loops is defined as positive. In contrast, when the change in the MO signal while reducing the field to 0 mT exceeds the loop amplitude, the amplitude is defined as negative.

Figure S13 shows the pump-intensity dependence of the $\Delta\gamma^{dif}$ signal in the concentric geometry, measured for a magnetic field of 16 mT, over a long time range up to 3 ns. Importantly, the decay of this signal does not change above I_{th}, resembling the dynamics of $\Delta T/T$.

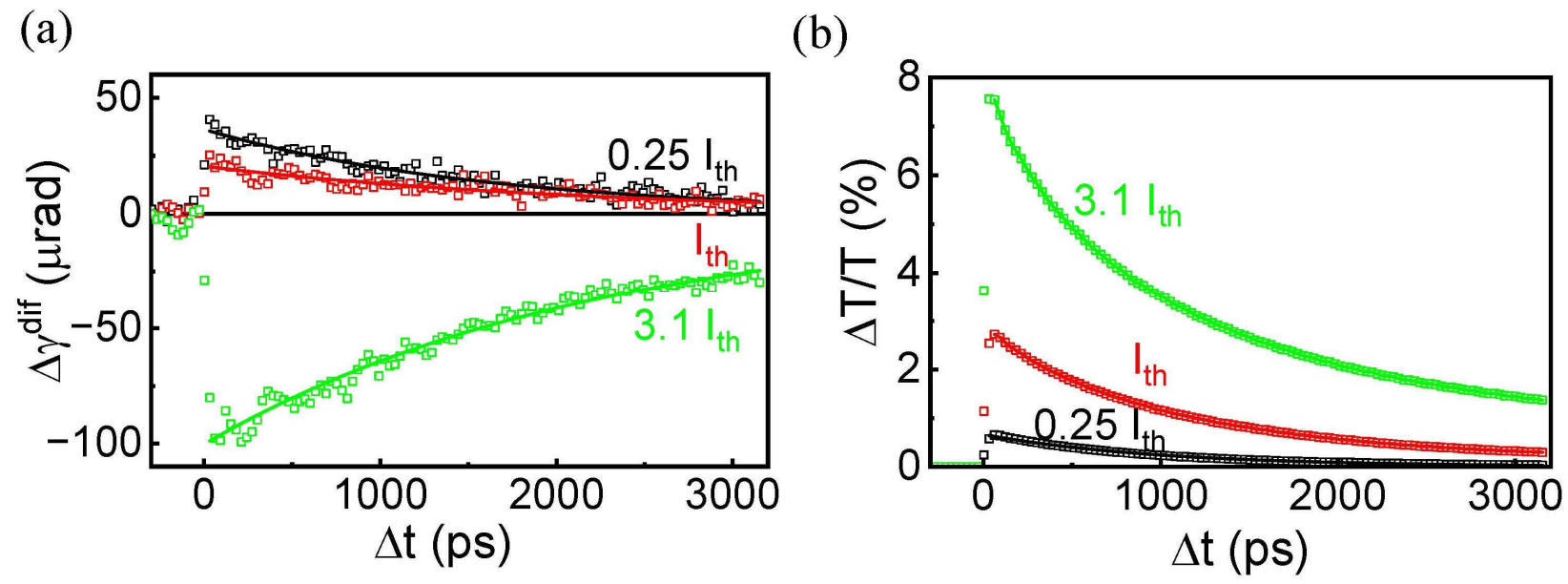

Fig. S13: Long time range dynamics measured in the concentric geometry. a, Ellipticity $\Delta\gamma^{dif}$ signal measured as a function of time delay for different pump intensities (points), lines are the fits using a monoexponential decay function plus background with the time constant of 1 ns. **b**, Dynamics of transient transmission $\Delta T/T$ measured for the same pump intensities (points), lines are the fits using a monoexponential decay function plus background with the time constant of 1 ns. Pump wavelength 800 nm, probe wavelength 532 nm.

Supplementary Note 3. Comparison of results measured in (Ge,Mn)Te with results reported for (Ga,Mn)As

In this Supplementary Note, we compare in detail the pump-induced MO signals observed in thin films of (Ge,Mn)Te with results reported for thin films of (Ga,Mn)As, which is the most thoroughly studied diluted magnetic semiconductor [S3].

In semiconductors, absorption of an intense femtosecond laser pulse photoexcites populations of excess electrons and holes, which thermalize within ≈ 100 fs through various scattering processes, forming local Fermi-Dirac distributions in the conduction and valence bands, respectively. Because the pump photon energy used in our experiment, 1.24–3.1 eV, is much larger than the indirect band gap of (Ge,Mn)Te (0.85 eV in GeTe [S4]), the excess energy of the photoexcited carriers is dissipated to the lattice by electron–phonon and hole–phonon scattering processes on a (sub)picosecond time scale. This creates an excess concentration of electrons and holes in the material. Since both (Ga,Mn)As [S3] and (Ge,Mn)Te [S5] are *p*-type semiconductors, the lifetime of photoexcited free electrons is much shorter than that of photoexcited free holes because traps for holes in the material are already filled by the background holes. When the photoexcited electrons are trapped at defect states, they transfer their energy to the lattice, leading to a temperature increase. Non-radiative recombination of photoexcited free holes with the trapped electrons increases the lattice temperature even further. Finally, the increased lattice temperature of the photoexcited semiconductor film relaxes back to its base temperature by heat diffusion to the sample substrate.

Unlike in (Ga,Mn)As [S6–S8], we used pump photon energies much larger than the band gap energy of (Ge,Mn)Te, resulting in high excess energies of the photoexcited carriers. These excess energies dissipate into the lattice through carrier–phonon scattering processes on a (sub)picosecond timescale. In our experiments, the pump wavelength was varied from 1000 nm (1.24 eV) to 400 nm (3.1 eV). Although the total amount of dissipated heat changes substantially between these wavelengths, the measured MO signals do not change significantly, as shown in Supplementary Figure S8 (see also Figure 5f). This rules out excess-energy dissipation–induced lattice heating as the dominant mechanism leading to magnetization precession in (Ge,Mn)Te. Consequently, trapping of free photoelectrons by defect centers is the fastest effect leading to a transient increase in the lattice temperature, which can, in principle, be responsible for the observed magnetization precession. This temperature increase builds up on a timescale of ≈ 10 ps and persists for ≈ 1 000 ps in (Ga,Mn)As [S7], and similar timescales are observed in the GeMnTe sample as well; see Supplementary Figure S9c and Figure S13 for experimental data illustrating the initial stages of the carrier dynamics and the signal decay over a nanosecond time range, respectively. As the magnetism in (Ge,Mn)Te is hole-mediated, the other effect, which can lead to pump-induced changes in the magnetic properties, is the excess density of photoholes in the valence band. There are several approaches by which these two contributions can be identified.

The first type of experimental data, in which thermal and nonthermal excitation mechanisms can be distinguished experimentally, is pump-induced precession of magnetization, which has been studied extensively in Ga_{1-x},Mn_xAs [S6–S8]. In this material, thermally induced precession of magnetization is observed in samples with low Mn doping ($x < 7\%$; see Figure 5 in Ref. S7) at relatively low pump fluences ($I < 70$ μJ cm^{-2} for $x = 3\%$; see Figure 2 in Ref. S7), where the position of the easy axis is determined by competing biaxial and uniaxial magnetic anisotropies that scale with magnetization as M^4 and M^2, respectively [S7,S8]. When non-radiative recombination of photoelectrons produces a transient increase in the lattice temperature, M is reduced and, consequently, the uniaxial anisotropy is enhanced relative to the biaxial anisotropy. This leads to a transient tilt of the easy axis orientation in the material, around which the Mn moments start to precess [S7]. The key fingerprint of this mechanism is the

relatively slow rise time of the MO signal, which develops over tens of picoseconds (see Figure 2 in Ref. S7) due to lattice heating resulting from photoelectron trapping [S7].

For higher Mn doping or higher fluences, the spin-orbit torque due to excess hole density in the spin-orbit-coupled, exchange-split valence band becomes the dominant excitation mechanism [S7]. The increased number of occupied hole states (compared with the equilibrium state in the dark) can generate a nonequilibrium hole spin polarization that is misaligned with the equilibrium orientation of the Mn moments. This non-equilibrium photohole polarization persists over the timescale of hole recombination, during which it exerts a torque on the Mn local moments. Experimentally, it appears as an impulsive tilt of the magnetization, which makes it possible to distinguish the spin-orbit torque from the considerably slower thermal excitation mechanism (see Figure 2 in Ref. S7).

In (Ge,Mn)Te, the oscillatory MO signal is fully developed in less than ≈ 1 ps after absorption of the pump pulse (see the inset in Figure 5c and Supplementary Note 2.8 for a detailed discussion). Therefore, the transient temperature increase on a timescale of ≈ 10 ps (Supplementary Figure S9c) cannot cause the magnetization precession in the (Ge,Mn)Te sample. The rapid energy transfer from photoexcited carriers to phonons (and magnons) also generates thermal stress in the material, which can launch coherent acoustic phonon wave packets (strain waves) and induce magnetization precession [S9]. However, because the duration of the induced strain pulses is on the order of tens of picoseconds (see Figure 1b in Ref. S9), this mechanism is again not compatible with the ultrafast process that triggers magnetization precession on the sub-picosecond timescale. This leaves the pump-induced excess hole density in the valence band, which creates a non-equilibrium hole spin polarization misaligned with the equilibrium orientation of the Mn moments and acts as a spin-orbit torque [S7], as the most probable cause of the observed impulsive tilt of the magnetization, leading to magnetization precession in the (Ge,Mn)Te sample.

The second type of experimental data, in which thermal and non-thermal excitation mechanisms can be distinguished experimentally, is the pump-induced change in hysteresis loops. A pump-induced temperature increase suppresses magnetic ordering in the ferromagnet (i.e., demagnetization), resulting in a reduction of its MO response [S10]. In contrast, a pump-induced enhancement of the MO signal was observed in the (Ge,Mn)Te sample, as evident from the identical sign of the measured hysteresis loops in the static experiment (Figure 2d) and in the low-intensity dynamical data (Figure 1e and Figure 6d). This MO signal enhancement can be attributed to an increase in hole concentration, which strengthens the hole-mediated magnetism in (Ge,Mn)Te [S5], as observed in (Ga,Mn)As [S11]. Moreover, the magnetic-field dependence of the MO signals becomes strongly non-monotonic for laser fluences approaching I_{th} (see Supplementary Figure S12), whereas the static hysteresis loops remain unchanged (i.e., monotonic functions of the external field) at all temperatures (Figure 2d). We attribute this effect, which is absent in (Ga,Mn)As, to the action of spin–orbit torque associated with the laser-induced change in the Rashba spin–orbit interaction, leading to a reconfiguration of the correlated spin-glass structure of the GeMnTe sample.

Supplementary references

[S1] D. Kriegner et al., Phys. Rev. B 94, 054112 (2016).
[S2] B. Koopmans, M. van Kampen, J. T. Kohlhepp, and W. J. M. de Jonge, Phys. Rev. Lett. 85, 844–847 (2000).
[S3] T. Jungwirth et al., Rev. Mod. Phys. 86, 855–896 (2014).
[S4] G. Kremeret et al., Phys. Rev. B 111, 235109 (2025).
[S5] Y. Fukuma et al., Appl. Phys. Lett. 94, 269901 (2009).
[S6] E. Rozkotová et al., Appl. Phys. Lett. 92, 122507 (2008).
[S7] N. Tesařová et al., Nature Phot. 7, 492–498 (2013).
[S8] P. Němec et al., Nature Commun. 4, 1422 (2013).
[S9] M. Bombeck et al., Phys. Rev. B 85, 195324 (2012).
[S10] A. Kirilyuk, A. V. Kimel, and T. Rasing, Rev. Mod. Phys. 82, 2731 (2010).
[S11] J. Wanget al., Phys. Rev. Lett. 98, 217401 (2007).
